# Deoxycholic Acid-Mediated Sphingosine-1-Phosphate Receptor 2 Signaling Exacerbates DSS-Induced Colitis through Promoting Cathepsin B Release

**DOI:** 10.1155/2018/2481418

**Published:** 2018-05-09

**Authors:** Shengnan Zhao, Zizhen Gong, Xixi Du, Chunyan Tian, Lingyu Wang, Jiefei Zhou, Congfeng Xu, Yingwei Chen, Wei Cai, Jin Wu

**Affiliations:** ^1^Department of Pediatric Surgery, Xinhua Hospital, School of Medicine, Shanghai Jiaotong University, Shanghai, China; ^2^Shanghai Institute for Pediatric Research, School of Medicine, Shanghai Jiaotong University, Shanghai, China; ^3^Shanghai Key Laboratory of Pediatric Gastroenterology and Nutrition, Shanghai, China; ^4^State Key Laboratory of Proteomics, National Center for Proteomics Science (Beijing), Beijing Institute of Radiation Medicine, Beijing, China; ^5^National Engineering Research Center for Protein Drugs, Beijing, China; ^6^Shanghai Institute of Immunology, Institutes of Medical Sciences, Shanghai Jiaotong University School of Medicine, Shanghai, China

## Abstract

We recently have proved that excessive fecal DCA caused by high-fat diet may serve as an endogenous danger-associated molecular pattern to activate NLRP3 inflammasome and thus contributes to the development of inflammatory bowel disease (IBD). Moreover, the effect of DCA on inflammasome activation is mainly mediated through bile acid receptor sphingosine-1-phosphate receptor 2 (S1PR2); however, the intermediate process remains unclear. Here, we sought to explore the detailed molecular mechanism involved and examine the effect of S1PR2 blockage in a colitis mouse model. In this study, we found that DCA could dose dependently upregulate S1PR2 expression. Meanwhile, DCA-induced NLRP3 inflammasome activation is at least partially achieved through stimulating extracellular regulated protein kinases (ERK) signaling pathway downstream of S1PR2 followed by promoting of lysosomal cathepsin B release. DCA enema significantly aggravated DSS-induced colitis in mice and S1PR2 inhibitor as well as inflammasome inhibition by cathepsin B antagonist substantially reducing the mature IL-1*β* production and alleviated colonic inflammation superimposed by DCA. Therefore, our findings suggest that S1PR2/ERK1/2/cathepsin B signaling plays a critical role in triggering inflammasome activation by DCA and S1PR2 may represent a new potential therapeutic target for the management of intestinal inflammation in individuals on a high-fat diet.

## 1. Introduction

High-fat diet (HFD) increases fecal bile acid level, especially secondary bile acid deoxycholic acid (DCA), which is considered to be associated with the development of inflammatory bowel disease (IBD) [1–5]. Prolonged high level of DCA in the gut could disrupt epithelial integrity [6, 7] and induce obvious intestinal inflammation and injury that resembles human IBD [8, 9]; however, the molecular mechanism underlining the triggering of inflammatory response by DCA remains unelucidated. Our previous studies show that high-level DCA could act as an endogenous damage-associated molecular pattern (DAMP) to induce NLRP3 inflammasome activation and proinflammatory cytokine-IL-1*β* production in macrophages, thus contributing to the HFD-related colonic inflammation [10].

Bile acids play their roles through specific receptors, including nuclear receptors [farnesoid X receptor (FXR), vitamin D receptor (VDR), and pregnane X receptor (PXR)] as well as membrane receptors [G-protein-coupled bile acid receptor 5 (TGR5), sphingosine-1-phosphate receptor 2 (S1PR2), and muscarinic receptor 2] [11–18], and may activate multiple signaling pathways [protein kinase B (AKT), extracellular regulated protein kinases (ERK1/2), and c-Jun N-terminal kinase (JNK)] [19–22]. We found that *in vitro* blockage of S1PR2 effectively prevented DCA-induced mature IL-1*β* secretion, indicating the effect of DCA on inflammasome activation is mainly mediated through S1PR2; however, the downstream signaling cascades involved in this process remain elusive and whether S1PR2 inhibition could ameliorate DCA-induced colonic inflammation need to be investigated.

Here, we show that DCA can dose-dependently up-regulate S1PR2 expression. The activation of NLRP3 inflammasome by DCA is mainly achieved through stimulating extracellular regulated protein kinases (ERK) signaling pathway downstream of S1PR2, and followed by promoting of cathepsin B release. DCA enema strongly aggravates DSS-induced colitis in mice and S1PR2 antagonist as well as inhibition of cathepsin B substantially alleviate colonic inflammation imposed by DCA.

## 2. Materials and Methods

### 2.1. Reagents

Deoxycholic acid (DCA), lipopolysaccharide (LPS), CA-074 Me, and JTE-013 were purchased from Sigma-Aldrich (St. Louis, MO, USA). Dextran sulfate sodium (DSS, molecular weight 36–50 kDa) was obtained from MP Biomedicals Inc. (Irvine, CA, USA). ASC antibody was from Santa Cruz Biotechnology (Santa Cruz, CA, USA). IL-1*β*, ERK, AKT antibodies, and U0126 were purchased from Cell Signaling Technologies (Beverly, MA, USA). DMEM and RPMI 1640 were obtained from Invitrogen (Carlsbad, CA, USA). ELISA Kits were from eBioscience (San Diego, CA, USA).

### 2.2. Animals

Six- to eight-week-old C57BL/6 female mice were purchased from Experimental Animal Center of the Chinese Academy of Sciences (Shanghai, China) and maintained on a 12 hr light/dark cycle in the specific pathogen-free animal facility. All the experimental procedures were approved by the Shanghai Laboratory Animal Care and Use Committee.

### 2.3. Cells

The murine macrophage cell line J774A.1 was obtained from Type Culture Collection of the Institutes of Biomedical Sciences, Fudan University (Shanghai, China). The cells were cultured in DMEM (Invitrogen) supplemented with 10% heat-inactivated fetal bovine serum (Gibico) and 1% penicillin/streptomycin (Invitrogen) at 37°C with 5% CO_2._ Peritoneal macrophages were isolated by peritoneal lavage with 2 ml PBS. Cells were collected and incubated for 3 hours at 37°C to remove nonadherent cells. The remaining adherent macrophages were used in experiments.

### 2.4. IL-1*β* Detection

J774A.1 cells were treated with LPS (1 *μ*g/ml) for 5 h before DCA (100 *μ*M) stimulation in the presence or absence of different inhibitors (e.g., JTE-013 and U0126), then supernatants were harvested and the release of bioactive IL-1*β* was determined by Western blot and ELISA according to the manufacturer's instructions (eBioscience).

### 2.5. ASC Speck Visualization

J774A.1 cells were fixed in paraformaldehyde (PFA, 4%) for 15 min and permeabilized with Triton-X 100 (0.1%) for 10 min. Cells were then incubated with ASC primary antibody for 1 hour, followed by Alexa Fluor 488-conjugated secondary antibody (Santa Cruz, CA, USA) and DAPI staining. Visualization of endogenous ASC was performed on a Leica fluorescence microscope.

### 2.6. Cathepsin B Imaging

LPS-primed macrophages were stimulated with or without DCA (100 *μ*M, 24 h) in the presence or absence of different inhibitors (JTE-013 and U0126), then the cells were incubated with cathepsin B fluorogenic substrate z-Arg-Arg cresyl violet (Neuromics) for 1 hour at 37°C, followed by Hoechst staining.

### 2.7. Real-Time PCR

Preparation of RNA and cDNA was performed using a Pure Yield RNA Midi-prep kit (Promega, WI, USA) and Transcriptor First Strand cDNA Synthesis Kit (Roche, NJ, USA). The *S1PR2* mRNA expression was evaluated by real-time PCR on the PikoReal Real-Time PCR System (Thermo, Waltham, MA, USA), using the following primers: forward 5′- ATGGGCGGCTTATACTCAGAG -3′ and reverse 5′- GCGCAGCACAAGATGATGAT -3′.

### 2.8. Western Blot

Macrophages were lysed by protein lysis buffer (Sigma-Aldrich) supplemented with protease and phosphatase inhibitors, and for the detection of released IL-1*β,* the cell culture supernatant was concentrated by acetone precipitation. Cell lysates or concentrated supernatant proteins were resolved by SDS-PAGE, transferred to 0.2 *μ*m polyvinylidene difluoride membranes (Millipore, MA, USA), and probed with antibodies against IL-1*β* and *β*-actin (Sigma-Aldrich). For the detection of phosphorylated ERK and AKT, LPS-primed macrophages were incubated with or without DCA (100 *μ*M) for indicated time point, and cell lysates were prepared and immunoblots were performed using specific antibodies against phospho-ERK (p-ERK) or p-AKT. The detection of cytosolic cathepsin B was performed as described previously [10]. In brief, J774A.1 cells were permeabilized with extraction buffer containing 50 *μ*g/ml digitonin for 15 min and the cell lysates were centrifuged. Then, the supernatants were collected and subjected to immunoblotted for cathepsin B.

### 2.9. Cytosolic Cathepsin B Activity Assay

Cytosolic protein was prepared as described above, and cathepsin B activity was examined by a fluorometric assay kit according to the manufacturer's instruction (ApexBio). Briefly, each sample (100 *μ*g) was incubated with reaction buffer (50 *μ*l) and substrate Ac-RR-AFC (2 μl) at 37°C for 1 h, and free AFC (amino-4-trifluoromethyl coumarin) was detected by a fluorescence spectrophotometer (excitation wavelength = 400 nm and emission wavelength = 505 nm).

### 2.10. RNA Interference

J774A.1 cells were transfected with S1PR2 and cathepsin B small interfering RNA or scrambled siRNA by using TransIT-Jurkat (Mirus Bio, Madison, WI, USA). Cells were then used to perform further experiment after 24 hours. RNA oligonucleotides sequences were as follows: S1PR2, forward 5′- CCU CUA CAA AGC CCA CUA UTT-3′ and reverse 5′- AUA GUG GGC UUU GUA GAG GTT-3′; cathepsin B, forward 5′- GCU GAA GAC CUG CUU ACU UTT-3′ and reverse 5′- AAG UAA GCA GGU CUU CAG CTT-3′.

### 2.11. Colitis Induction and Cathepsin B Inhibition

Colitis was induced in C57BL/6 female mice with 2.5% DSS dissolved in drinking water for 7 days. Animals were randomly divided into three groups, receiving colorectal instillation of PBS, 4 mM DCA, and 4 mM DCA plus intraperitoneal injection of cathepsin B inhibitor (Ca-074Me, 10 mg/kg/day), respectively, for 7 consecutive days from day 1 of DSS treatment (*n* = 7 in each group). Body weight, stool consistency, and rectal bleeding were monitored daily. Mice were sacrificed and colon length was measured on day 8. The paraffin sections of colon tissues were stained with hematoxylin and eosin. Colon homogenates were collected to detect mature IL-1*β* and evaluate MPO activity.

### 2.12. In Vivo S1PR2 Suppression

To evaluate the role of S1PR2 in the colonic inflammation exacerbated by DCA, colitis was induced in C57BL/6 mice as described above and animals were randomly divided into four groups, including control group, DSS-treated group, DSS-treated plus DCA enema group, and DSS-treated plus DCA enema-S1PR2 inhibitor group (JTE-013, 10 mg/kg/day for 7 consecutive days from day 1 of DSS treatment, *n* = 7/each group). On day 8, mice were sacrificed for sample collection and analysis as mentioned above.

### 2.13. Statistics

Data was compared by using two-tailed Student's *t*-test or one-way analysis of variance (ANOVA). Results were expressed as mean ± standard error of the mean (s.e.m.). *p* values below 0.05 were deemed to be significantly different.

## 3. Results

### 3.1. DCA Upregulates S1PR2 Expression in Macrophages

**Figure 1:**
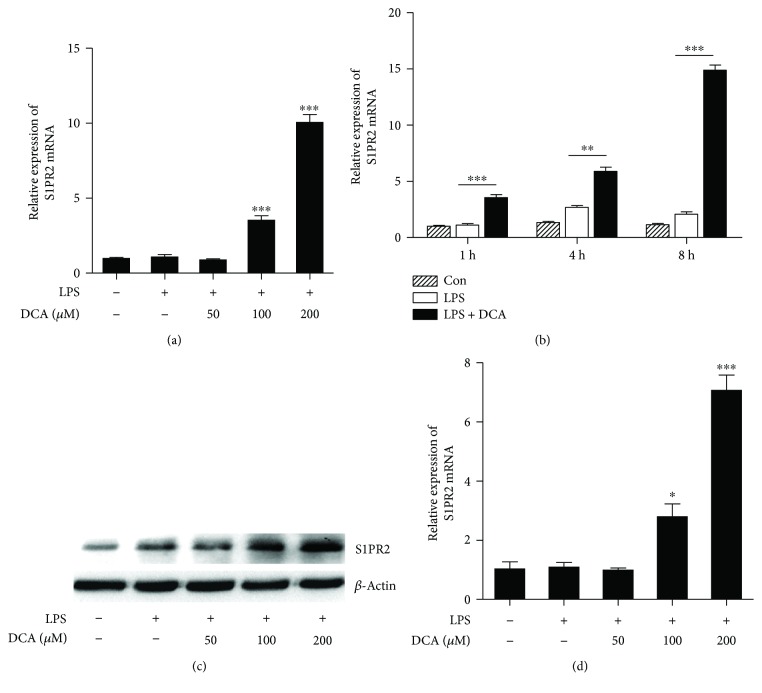
DCA induces S1PR2 expression in macrophages. (a) J774A.1 macrophages were pretreated with or without LPS and then stimulated with different dosage of DCA (0, 50, 100, and 200 *μ*M) for 4 h. Relative S1PR2 mRNA expression level was analyzed by real-time PCR. (b) J774A.1 macrophages were pretreated with or without LPS and then stimulated with DCA (100 *μ*M) for different time courses. Relative S1PR2 mRNA expression level was analyzed by real-time PCR. (c) J774A.1 macrophages were pretreated with or without LPS and then stimulated with different dosage of DCA (0, 50, 100, and 200 *μ*M) for 24 h. S1PR2 protein level was detected by Western blotting. (d) Real-time PCR determinant of relative S1PR2 mRNA expression in murine peritoneal macrophages treated with different dosage of DCA. ^∗^ *p* < 0.05, ^∗∗^ *p* < 0.01, and ^∗∗∗^ *p* < 0.001. Error bars indicate s.e.m. Representative data from 3 independent experiments giving similar results are shown.

The NLRP3 inflammasome is reported to recognize multiple endogenous danger signals and trigger the release of proinflammatory cytokines including active IL-1*β*, thus contributing to many diseases such as atherosclerosis and T2 diabetes [23–26]. To observe the effect of DCA on the expression of sphingosine-1-phosphate receptor 2 (S1PR2), LPS-primed macrophage cell line J774A.1 was incubated with different dosage of DCA, and the results showed that DCA could dose and time dependently upregulate the mRNA level of S1PR2 compared to the LPS-treated alone group (Figures 1(a) and 1(b)). The increase of S1PR2 protein level in response to DCA was further confirmed by Western blotting (Figure 1(c)). Meanwhile, DCA also exerted similar effects on murine peritoneal macrophages (Figure 1(d)). These data suggest that high-level DCA could induce S1PR2 expression in macrophages, thus initiating the activation of related downstream signaling, which may contribute to the cathepsin B release and inflammasome activation.

### 3.2. DCA Activates ERK Signaling and Promotes Cathepsin B Release

**Figure 2:**
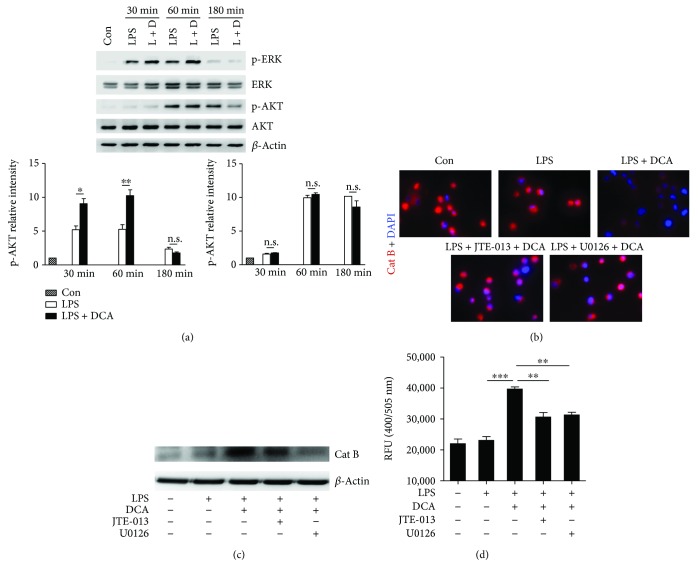
DCA activates ERK signaling and promotes cathepsin B release. (a) Immunoblot analysis of phospho-ERK, total ERK, phospho-AKT, and total AKT of LPS-primed J774A.1 macrophages treated with or without DCA (100 *μ*M) for different time courses. *β*-Actin was regarded as a loading control. The relative intensity of the bands was quantitated. (b) LPS-primed J774A.1 macrophages were treated with DCA (100 *μ*M) in the presence or absence of JTE-013 or U0126. The cells were incubated with cathepsin B substrate which emits a red signal upon cleavage by cathepsin B, followed by Hoechst staining (blue). (c–d) LPS-primed J774A.1 macrophages were treated with DCA (100 *μ*M) in the presence or absence of JTE-013 or U0126, and cytosolic protein was extracted for the immunoblot analysis of mature cathepsin B (c) as well as fluorometric assay of cathepsin B activity (d). ^∗^ *p* < 0.05, ^∗∗^ *p* < 0.01, and ^∗∗∗^ *p* < 0.001. Error bars indicate s.e.m. The data shown are from 3 independent experiments.

We sought to figure out the molecular mechanism underlying the induction of cathepsin B release mediated by S1PR2 in response to DCA. ERK1/2 and AKT pathways have been reported to be major signaling cascades downstream of S1PR2, and we found that DCA stimulation could obviously increase the phosphorylation of ERK1/2 instead of AKT in the LPS-primed macrophages (Figure 2(a)). As we reported previously, DCA induces NLRP3 inflammasome activation mainly through promoting cathepsin B release [10] and therefore DCA treatment significantly decreased the staining of lysosomal cathepsin B, while silencing S1PR2 expression with siRNA or S1PR2 antagonist (JTE-013) as well as ERK1/2 inhibitor (U0126) could dramatically prevent DCA-induced cathepsin B leakage from lysosome as evidenced by the increased lysosomal cathepsin B staining and decreased cytosolic cathepsin B level and activity (Supplementary Figure 1 and Figures 2(c)–2(d)). These results suggest that the induction effect of DCA on cathepsin B release in macrophages may be at least in part mediated through S1PR2/ERK signaling pathway.

### 3.3. Blockage of ERK Signaling Prevents ASC Speck Formation and Mature IL-1*β* Secretion

**Figure 3:**
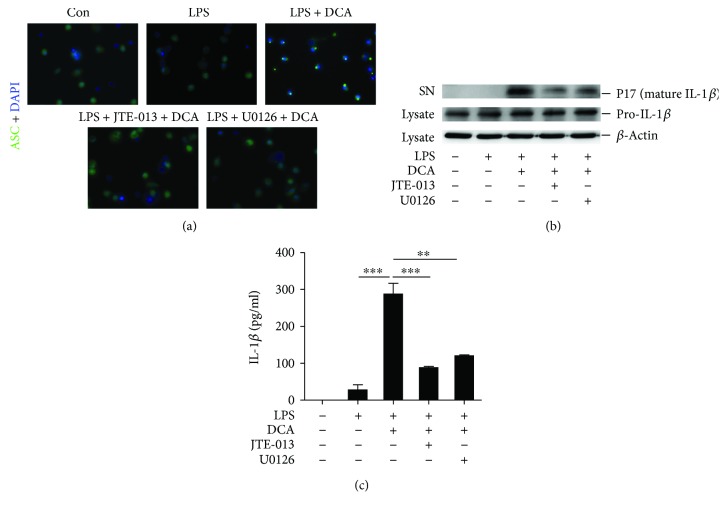
Blockage of ERK signaling prevents ASC speck formation and mature IL-1*β* secretion. (a) LPS-primed J774A.1 macrophages were treated with DCA in the presence or absence of JTE-013 or U0126. The cells were then stained with ASC antibody and DAPI. Representative immunofluorescent images of ASC specks were shown. (b) LPS-primed J774A.1 macrophages were treated with DCA in the presence or absence of JTE-013 or U0126 for 24 h. Immunoblot was performed to determine mature IL-1*β* (mIL-1*β*, 17kD) in the culture supernatants (SN) and precursors of IL-1*β* (pro-IL-1*β*) in cell lysates, and (c) secreted IL-1*β* in supernatants was also analyzed by ELISA. ^∗∗^ *p* < 0.01 and ^∗∗∗^ *p* < 0.001. Error bars indicate s.e.m. The data shown are representative of 3 independent experiments.

To further confirm the role of S1PR2/ERK signaling on the NLRP3 inflammasome activation induced by DCA, immunofluorescent staining of ASC specks was performed. Upon inflammasome activation, ASC assembles into a large protein complex termed “speck,” which serves as a platform to recruit procaspase-1 and promotes its autocatalytic activation, finally leading to the release of proinflammatory cytokines, such as IL-1*β*. Therefore, the formation of ASC speck can be regarded as a readout for inflammasome activation. Indeed, LPS-primed macrophages showed obvious ASC speck formation upon DCA stimulation, while pretreated with ERK inhibitor (U0126) or S1PR2 antagonist (JTE-013) as well as cathepsin B siRNA almost completely prevented ASC speck formation (Figure 3(a) and Supplementary Figure 2). Consistently, ERK or S1PR2 inhibition substantially reduced mature IL-1*β* production in macrophages exposed to DCA (Figures 3(b) and 3(c)). These data illuminate that S1PR2/ERK signaling pathway may be involved in the DCA-induced inflammasome activation.

### 3.4. DCA Administration Exacerbates DSS-Induced Colitis and Inhibition of Cathepsin B Release Reverses the Intestinal Inflammation

**Figure 4:**
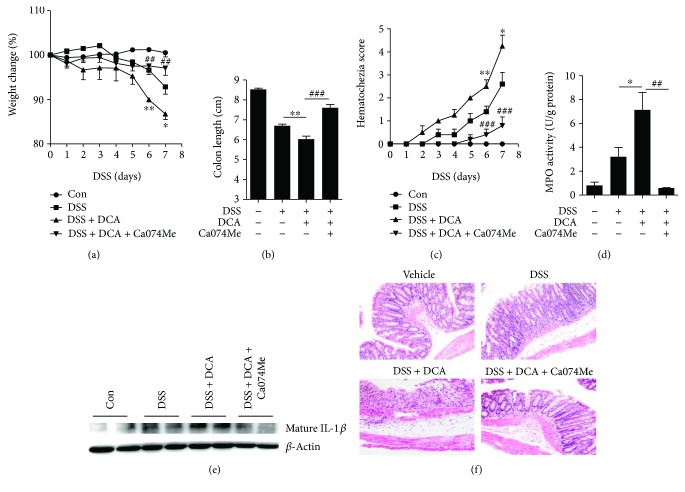
DCA administration exacerbates DSS-induced colitis and inhibition of cathepsin B release reverses the intestinal inflammation. Colitis was induced in mice with 2.5% DSS and animals were divided into control, DSS-treated, DSS-treated plus DCA enema, DSS-treated plus DCA enema, and cathepsin B inhibitor (Ca-074Me)-injection groups. (a) Loss of basal body weight, (b) colon length, (c) hematochezia score, and (d) MPO activity in colon tissue were detected. (e) Immunoblot analysis of mature IL-1*β* (17kD) in colonic homogenates. (f) HE staining of distal colon sections of differently treated mice. ^∗^ *p* < 0.05 and ^∗∗^ *p* < 0.01 compared to the DSS-treated alone mice. ^#^^#^ *p* < 0.01 and ^#^^#^^#^ *p* < 0.001 compared to the DSS-treated plus DCA enema mice. Error bars indicate s.e.m. The data shown are from 3 individual experiments.

As we reported previously, DCA induces NLRP3 inflammasome activation mainly via promoting cathepsin B release and colorectal instillation of DCA could significantly exacerbate DSS-induced colitis through activating NLRP3 inflammasome [10]. To investigate whether inhibition of cathepsin B release could ameliorate the effect of DCA instillation, DSS-treated mice received an enema of PBS, DCA, or DCA plus cathepsin B inhibitor (Ca074Me) administration. In line with our previous findings, colorectal DCA instillation caused much more severe inflammation and injury than DSS treatment alone (Figures 4(a)–4(f)), whereas inhibition of cathepsin B potently decreased the colonic mature IL-1*β* level and significantly restrained the aggravating role of DCA in the DSS-induced colitis, as evidenced by much less body weight loss and colon length shortening as well as lower MPO activity and hematochezia score compared to the DCA enema group (Figures 4(a)–4(f)). These data offer further *in vivo* evidence that cathepsin B contributes to the DCA-induced inflammasome activation.

### 3.5. S1PR2 Antagonist Abrogates the Deteriorating Role of DCA in the DSS-Induced Colitis

**Figure 5:**
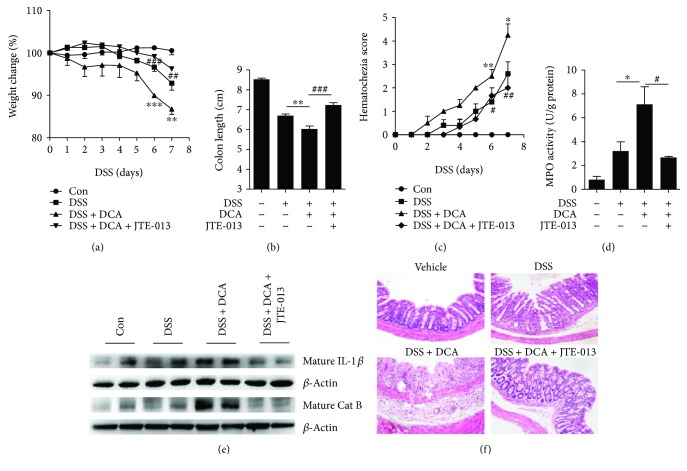
S1PR2 antagonist abrogates the exacerbating role of DCA in the DSS-induced colitis. (a) Loss of basal body weight, (b) colon length, (c) hematochezia score, and (d) MPO activity in colon tissue of control, DSS-treated, DSS-CL, DSS-treated plus DCA enema, and DSS-treatedplus DCA enema, and S1PR2 antagonist (JTE-013)-injection mice. (e) Immunoblot analysis of mature IL-1*β* (17kD) and mature cathepsin B in colon tissue. (f) HE staining and of distal colon sections of differently treated mice as described above. ^∗^ *p* < 0.05, ^∗∗^ *p* < 0.01, and ^∗∗∗^ *p* < 0.001 compared to the DSS-treated alone mice. ^#^ *p* < 0.05, ^#^^#^ *p* < 0.01, and ^#^^#^^#^ *p* < 0.001 compared to the DSS-treated plus DCA enema mice. Error bars indicate s.e.m. Representative data from 3 individual experiments are shown.

We observed that high-level DCA could activate inflammasome via regulating the expression of bile acid receptor-S1PR2 in macrophages, which may be at least partially responsible for the HFD-related intestinal inflammation. We sought to study whether blockage of S1PR2 could prevent the proinflammatory role of DCA instillation in DSS-induced colitis; thus, S1PR2 antagonist JTE-013 was intraperitoneally administrated. The results showed that S1PR2 blockage significantly reduced the body weight loss and colon length shortening (Figures 5(a) and 5(b)), exhibiting much lower MPO activity and hematochezia score (Figures 5(c) and 5(d)), meanwhile ameliorating mucosal inflammatory cell infiltration and mucosal epithelium disruption (Figure 5(f)). More importantly, S1PR2 blockage dramatically reduced mature cathepsin B level and mature IL-1*β* production in colon tissues caused by DCA instillation (Figure 5(e)), corroborating the *in vitro* findings that DCA induces inflammasome activation and proinflammatory cytokine secretion mainly through the S1PR2 pathway.

## 4. Discussion

A westernized high-fat diet is closely related to the development of inflammatory bowel disease (IBD). HFD could increase fecal DCA level which subsequently contributes to the NLRP3 inflammasome activation and intestinal inflammation. Elucidation of the detailed mechanism is beneficial to provide a new therapeutic strategy for IBD treatment. In this study, we proved that DCA could dose dependently regulate the expression of S1PR2, the major bile acid receptor that mediated the effect of DCA on inflammasome activation in macrophages. Excessive DCA-triggered NLRP3 inflammasome activation and bioactive IL-1*β* production involved in the upregulation of ERK1/2 signaling, which resulted in the lysosomal cathepsin B release. *In vivo* experiments showed that, consistent with our previous studies, DCA colorectal instillation at the concentration comparable to HFD obviously induced IL-1*β* production and aggravated DSS-induced colitis, whereas blockage of S1PR2 signaling or inhibition of cathepsin B effectively protected mice from the exacerbated inflammation superimposed by DCA, as evidenced by much less body weight loss, significantly ameliorated colitis severity, and importantly, pronounced reduction of bioactive IL-1β level in colon tissue. Thus, our results suggest a possible molecular mechanism through which high-level DCA triggers colonic inflammation.

The multiple functions of bile acids are mediated by a variety of receptors [19]. S1PR2, as a G-protein coupled receptor, has been reported to express in monocytes/macrophages and also be required for degranulation and cytokine release by activated mast cells [27]; thus, its role in diverse inflammatory disorders has been gradually recognized. Zhang and colleagues characterized S1PR2 as a novel regulator of vascular inflammation that is critical in endothelial responses to injury during endotoxemia [28]. McMillin et al. emphasized that conjugated bile acid taurocholic acid- (TCA-) mediated S1PR2 signaling could promote neuroinflammation during hepatic encephalopathy [29]. Additionally, S1PR2 has been also implicated in modulating proinflammatory cytokine production in inflammatory bone loss diseases as well as atherosclerosis [30, 31]. Together with our data showing that S1PR2 mediated high-level DCA-induced intestinal inflammation, these findings provide a strong basis for pharmaceutical studies to verify the efficacy of S1PR2 antagonists in the treatment of inflammatory diseases, including IBD. Furthermore, our study indicates that ERK1/2 signaling is critical in the S1PR2-mediated cathepsin B release and subsequent NLRP3 inflammasome activation. As an important downstream pathway of S1PR2, ERK1/2 signaling has been involved in many physiological and pathological processes induced via S1PR2. In primary rodent hepatocytes, bile acids can activate ERK1/2 and AKT signaling pathways through S1PR2 and thereby play an important role in the regulation of hepatic glucose and lipid metabolism [17, 32, 33]. In another aspect, bile duct obstruction (BDO) has been reported to promote cholangiocarcinoma (CCA) progression, and evidence showed that elevated levels of conjugated bile acids activated ERK1/2 signaling pathway and were partially responsible for the cholangiocarcinoma cell growth and invasion, which could be significantly inhibited by S1PR2 antagonist [22]. Therefore, the function of S1PR2/ERK1/2 signaling may vary in different cell types and the target genes of this pathway in macrophages should be further investigated.

Moreover, S1PR2 activation is also found to be involved in the disruption of endothelial barrier structure and function. Sanchez et al. reported that activation of S1PR2 in endothelial cells could result in Rho-ROCK-PTEN pathway dependent destruction of adherens junctions and enhancement of paracellular permeability, thus leading to the increased vascular permeability [34]. Multiple sclerosis is an inflammatory disease which is characterized by the blood-brain barrier (BBB) dysfunction. In an *in vitro* BBB model enhanced S1PR2 signaling altered adherens junction formation at least partially via activation of Rho-ROCK pathways [35]. Furthermore, Rho-ROCK signaling activation also participated in the ethanol-induced disruption of intestinal epithelial tight junction integrity [36]. Considering numerous studies showing that high-level DCA is toxic to intestinal epithelium and able to increase intestinal permeability, together with our findings that DCA can regulate the expression of S1PR2, these data indicate that S1PR2 signaling may be also involved in DCA-induced intestinal barrier dysfunction, whereas the detailed molecular mechanisms still need to be confirmed.

## 5. Conclusions

Collectively, high-level DCA induced by HFD contributes to the development of IBD, and our data reveal a possible molecular mechanism and highlight the critical role of S1PR2/ERK1/2 signaling in triggering inflammasome activation. Therefore, targeting this pathway may represent a promising strategy to alleviate intestinal inflammation especially in individuals on a high-fat diet.

## Supplementary Materials

Supplementary Materials Figure 1: S1PR2 knockdown prevents DCA-dependent cathepsin B release. S1pr2 siRNA (siS1pr2) or control siRNA (siCTL) transfected J774A.1 macrophages were primed with LPS and then treated with DCA (100 *μ*M). The cells were incubated with cathepsin B substrate which emits a red signal upon cleavage by cathepsin B, followed by Hoechst staining (blue). Supplementary Figure 2: cathepsin B silencing inhibits ASC speck formation induced by DCA. Cathepsin B siRNA (siCat B) or control siRNA (siCTL) transfected J774A.1 macrophages were primed with LPS and then treated with DCA (100 *μ*M). The cells were subsequently stained with ASC antibody (green) and DAPI.
