# Supplementary material for: Deoxycholic Acid-Mediated Sphingosine-1-Phosphate Receptor 2 Signaling Exacerbates DSS-Induced Colitis through Promoting Cathepsin B Release
Source: J Immunol Res. 2018 May 9;2018:2481418. doi: 10.1155/2018/2481418 (PMC5966668; doi:10.1155/2018/2481418)
Supplement: Supplementary Materials — Figure 1: S1PR2 knockdown prevents DCA-dependent cathepsin B release. S1pr2 siRNA (siS1pr2) or control siRNA (siCTL) transfected J774A.1 macrophages were primed with LPS and then treated with DCA (100 μM). The cells were incubated with cathepsin B substrate which emits a red signal upon cleavage by cathepsin B, followed by Hoechst staining (blue). Supplementary Figure 2: cathepsin B silencing inhibits ASC speck formation induced by DCA. Cathepsin B siRNA (siCat B) or control siRNA (siCTL) transfected J774A.1 macrophages were primed with LPS and then treated with DCA (100 μM). The cells were subsequently stained with ASC antibody (green) and DAPI. [file 2481418.f1.doc]

**Supplementary Data**

**
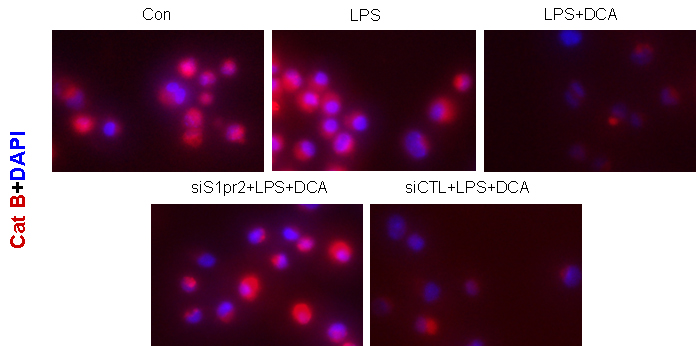
**

**Supplementary Figure 1. S1PR2 knockdown prevents DCA-dependent cathepsin B release.** S1pr2 siRNA (siS1pr2) or Control siRNA (siCTL) transfected J774A.1 macrophages were primed with LPS and then treated with DCA (100 μM). The cells were incubated with cathepsin B substrate which emits red signal upon cleavage by cathepsin B, followed by Hoechst staining (blue).


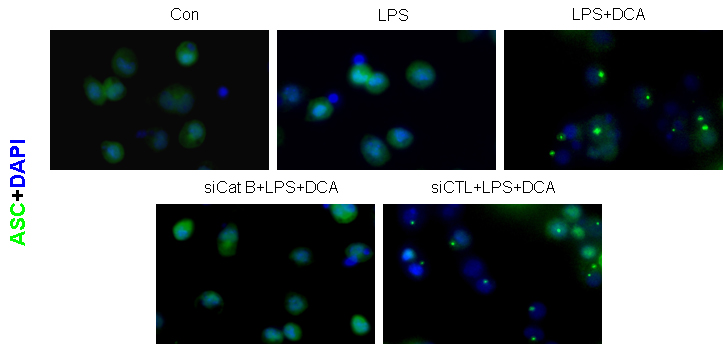


**Supplementary Figure 2. Cathepsin B silencing inhibits ASC specks formation induced by DCA.** Cathepsin B siRNA (siCat B) or Control siRNA (siCTL) transfected J774A.1 macrophages were primed with LPS and then treated with DCA (100 μM). The cells were subsequently stained with ASC antibody (green) and DAPI.
